# circACTG1 Promotes Hepatocellular Carcinoma Progression by Regulating miR-940/RIF1 Axis and Activating AKT/mTOR Pathway

**DOI:** 10.1155/2022/8649386

**Published:** 2022-06-19

**Authors:** Chunchen Wu, She Tian, Yuting Guo, Chao Yu, Linhan Lei, Dijie Zheng, Huahua Xie, Jian Zheng, Chengyi Sun

**Affiliations:** ^1^Guizhou Medical University, Guiyang 550004, China; ^2^Department of Hepatobiliary Surgery, The Affiliated Hospital of Guizhou Medical University, Guiyang 550004, China; ^3^Soochow University, Suzhou 2150002, China; ^4^Department of Hepatic-Biliary-Pancreatic Surgery, The Affiliated Hospital of Guizhou Medical University, Guiyang 550000, China; ^5^Key Laboratory of Liver, Gallbladder, Pancreas and Spleen of Guizhou Medical University, Guiyang 550004, China

## Abstract

**Background:**

Hepatocellular carcinoma (HCC) is recognized as the fourth in incidence and the third in mortality worldwide. The onset of HCC is insidious and often asymptomatic at the early stage. HCC is more prone to metastasis, recurrence, and drug resistance than other solid tumors owing to its feature of high heterogeneity. Therefore, what particularly important is to search for effective molecular markers in the occurrence and progression of HCC.

**Aim:**

To probe into the therapeutic potential of circACTG1 (hsa_circ_0046144) in HCC cell migration and invasion, providing a new insight and molecular target to diagnose and cure HCC patients.

**Methods:**

The circACTG1 expression in collected HCC cells was determined by quantitative polymerase chain reaction (qPCR). Assessment for circACTG1 diagnosing capability was analyzed by receiver operating characteristic (ROC) curves. Transwell assay, wound healing assay, and cell counting kit-8 assay were used for monitoring the effect of circACTG1 in HCC cell invasion, migration, and proliferation, respectively; qPCR, luciferase reporter assay, databases, and Western blot analysis were used for identifying the modulation mechanisms among circACTG1, miRNA-940, and RIF1. What is more, our study verified AKT-mTOR signaling after miR-940 mimic treatment or circACTG1 knockdown.

**Results:**

circACTG1 was overexpressed in HCC cells and tissues. Knockdown of circACTG1 restrained 97H and Huh7 cell migration and invasion. Significantly, circACTG1 was discovered to serve as a miR-940 sponge. miR-940 activation rebated the circACTG1 level, and conversely, miR-940 inhibition boosted the circACTG1 level. However, this effect or relationship was not seen after circACTG1 mutation. Furtherly, miR-940-downregulated expression was also found in HCC patients, and importantly, miR-940 inhibition reversed circACTG1 expression in 97H cells with circACTG1 knockdown. Moreover, the expression of RIF1 was significantly reduced after inhibiting circACTG1 or overexpressing miR-940 but rescued when both circACTG1 and miR-940 were inhibited. Finally, circACTG1 and miR-940 played significant roles of regulating AKT-mTOR signaling.

**Conclusion:**

circACTG1 expression remarkably ascended in HCC, which is of certain diagnostic value. Moreover, circACTG1 potentially regulates HCC cell proliferation, invasion, and migration via miR-940/RIF1/AKT/mTOR pathway.

## 1. Introduction

As the most frequent for primary liver cancer, hepatocellular carcinoma (HCC) accounts for almost 40% death in liver cancer all over the world [1]. Although recent studies have witnessed the development and improvement of surgery in HCC therapy, it is still a big challenge for revealing the underline mechanism of HCC due to its long incubation period, easy metastasis, and easy recurrence after operation [2]. Aside from surgery, it is not ideal for HCC patient prognosis by employing the more conventional treatments such as radiotherapy and chemotherapy. Fortunately, the rapid progress of some advance therapy methods in recent years, such as targeted therapy and immunotherapy[3–7], provides us a new enlightenment. The outcome for HCC treatment is encouraging, and we should strike while the iron is hot and delve deeper.

Circular RNAs (circRNAs) have been proved to be functional regulated elements in epigenetic silence, metabolite homeostasis, tumor progress, and other various bioprocesses [8–10]. Although there has been declared that circRNAs are heterogeneously expressed in many tumor cells [11], raising the possibility and potential role of circRNAs in diagnostic and prognosis, the evidence connecting circRNAs and HCC remains a mystery. Our research focused on the biologic function and mechanisms of circRNAs in cancer progress. Reviewing previous studies, we found that circRNAs act as a vital role in cancer occurrence and progression [8, 12]. Scholars have discovered the functions and molecular mechanisms of some circRNAs in the gastric cancer (GC) environment, which in turn affect the progression of GC, and these circRNAs have shown great potential as biomarkers for GC diagnosis or prognosis [13]. Zhang et al. discovered that circular RNA circNRIP1 promoted GC progression by sponging microRNA-149-5p via regulating the AKT1/mTOR pathway [14]. Coincidentally, in prostate cancer, some circRNAs can affect the metastasis and proliferation abilities of cancer cells, and their abnormal expression has an impact on the effect of chemotherapy or radiotherapy on the tumor [15]. Of course, in hepatocellular carcinoma, we also found that it can not only affect the process of liver cancer but also has great research value as a tumor marker and a therapeutic target [16].

Here, we firstly found that circACTG1, a significantly expressed circRNA in HCC comparing to adjacent normal tissues, promotes the HCC development through regulating miR-940/RIF1 axis and activating AKT/mTOR pathway. Our research gives a novel perspective on HCC pathogenesis and theoretical foundation for HCC therapy.

## 2. Materials and Methods

### 2.1. HCC Sample Collection

Tissue samples were obtained from HCC patients who were diagnosed between 2016 and 2018 at the Affiliated Hospital of Guizhou Medical University, after obtaining the participants' informed consent. In this study, the tissue samples verified by postoperative pathology were included. Each patient who was diagnosed with HCC was performed for long-term follow-up.

### 2.2. HCC Cell Line Culture and Transfection

A common liver cell line L02 and six HCC cell lines (SK-Hep1, HepG2, Hep3B, SMMC-7721, Huh7, and 97H) were included in the study. L02 and 97H were from American Type Culture Collection (Manassas, United States). SMMC-7721, SK-Hep1, HepG2, Huh7, and Hep3B were from Chinese Academy of Sciences' Cell Bank (Shanghai, China). All the above cell lines were confirmed via short tandem repeat profiling between 2019 and 2021. On the basis of ATCC protocols, the cells were cultivated in Dulbecco's Modified Eagle's medium with 1% penicillin/streptomycin and 10% fetal bovine serum (Gibco, Carlsbad, United States), maintaining at 37°C in a humidified incubator with 5% CO_2_. Routine mycoplasma was tested by PCR. The cell growth did not exceed 10 generations in total for each experiment. Lipo3000 transfection reagent (Invitrogen, Carlsbad, USA) was utilized for transfecting miR-940 mimic/inhibitor or negative control (NC, Ribobio, China; 200 nM) and short hairpin circACTG1 plasmid (shcircACTG1; GeneChem, China).

### 2.3. Cell Counting Kit-8 Assay

Cell counting kit-8 (CCK-8) assay was applied to test cell proliferation. Firstly, 1,000 suspended cells were cultivated in complete medium after being implanted on 96-hole plates. After the cells were adhered to the plates, the cell viability was determined at 0 h, 24 h, 48 h, and 72 h. Each hole was mixed with 180 *μ*L serum-free medium and then followed by 10 *μ*L CCK-8 assay reagent. With a microplate reader the absorbance value 2 h later at 450 nm was read and recorded.

### 2.4. Clone Formation Assay

Culture 97H cells for about 7-14 days with complete medium on 6-hole dishes at 1,000cell/hole, and then observe the formation of clones. Next, use 4% paraformaldehyde for 20 min fix and 0.1% crystal violet solution for 15 min staining. Finally, randomly select three fields to calculate the clone number and test the ability of cell proliferation.

### 2.5. Wound Healing Assay

Following transfection, cultivate 97H and Huh7 cells with culture solution (Ibidi, Germany) on a 6-hole plate at a density of 3 × 104 cells. Then, 24 h later, remove the culture solution and clean the cells with PBS twice. Each dish was added with 2 *μ*L of serum-free medium and the cells were forested for 48 h. Capture images, and measure the wound volume by applying ImageJ software (National Institutes of Health, United States).

### 2.6. Matrigel Invasion Assay

Six-hole dishes with 8-*μ*m chambers (Corning, United States) were applied for cellular invasion assessment (with Matrigel). Firstly, implant the transfected 97H and Huh7 cells on 6-hole dishes at 1 × 105 cell concentration. Add serum-free medium 200 *μ*L to the upper chamber and 800 *μ*L medium with 30% FBS to the lower chamber. Around 48 h later, fix the cells for 20 min in paraformaldehyde (4%) and stain for 15 min in crystal violet solution (0.1%). Randomly select 3 fields to calculate invasion cell number and to assess the cell invasion ability.

### 2.7. Western Blot Analysis

97H cell was lysed utilizing RIPA buffer with 1% phosphatase and protease inhibitor cocktail (Thermo Fisher Scientific). For cell lysates, separate with 10% SDS-PAGE first, transfer to PVDF membranes (Millipore) via electrophoresis, block in 5% nonfat milk in TBST, and incubate overnight at 4°C with indicated antibodies. Primary antibodies were from commercial sources: anti-RIF1 (Abcam, ab13422), anti-PI3K p85-*α*/*γ* (Affinity, AF6242), anti-p-PI3K p85-*α*/*γ* (Affinity, AF3242), anti-p-AKT (Cell Signaling Technology, #4060), anti-mTOR (Cell Signaling Technology, #2983), anti-AKT (Cell Signaling Technology, #4691), anti-*β*-actin (Abcam, ab6276), and anti-p-mTOR (Cell Signaling Technology, #5536) as the internal reference. Dilute the secondary antibodies against rabbit and mouse at 1 : 5000, culture the membranes for 1 h at room temperature, and visualize by chemiluminescence.

### 2.8. RNA Extraction and qPCR Analysis

Extract total RNA from tissues and cells with TRIzol™ Reagent (Thermo Fisher Scientific), and synthesize cDNA from total RNA (1 *μ*g) by PrimeScript™ RT Master Mix (Takara). Quantitative real-time PCR was performed by TB Green® Premix Ex Taq™ (Takara) on 7500HT Fast Real-Time PCR System (Applied Biosystems; Thermo Fisher Scientific). See Table 1 for the primers used. Normalize gene expression to *β*-actin and determine it by 2^−*ΔΔ*Ct^ method.

### 2.9. Dual-Luciferase Reporter Assay

Firstly, inoculate cells in a 24-hole dish and transfect with circACTG1 wildtype or circACTG1 mutant plasmid with or without miR-940 mimic/inhibitor. Two days later following transfection, harvest the cells and measure luciferase activity by the dual-luciferase reporter assay system (Promega).

### 2.10. *In Vivo* Subcutaneous Xenograft Mouse Models

Inoculated 97H cells (5 × 10^6^ cells in 0.1 mL culture medium) were transfected with circACTG1 knockdown (shcircACTG1) or normal control (shNC) and antagomiR-940 into 4 to 6-week-old male BALB/c nude mice on the left and right dorsal flanks, respectively (*n* = 5 each group). Measure tumor areas every 2 days with a caliper and utilize the formula, *W*^2^ × *L*/2 (*W* = the shortest diameters of the tumor and *L* = the longest diameters). After 30 days, sacrifice the mice and measure the tumor size and weight.

### 2.11. Statistical Analysis

Statistical analyses were processed with SPSS 25.0 software. Paired *t*-test was constructed to contrast circACTG1 mRNA level between tumor group and control group; *t*-test was used to analyze the difference between the two groups; one-way analysis of variance was used to compare means of ≥3 experimental groups. Pearson's correlation was applied for the correlation analysis between circACTG1 and miR-940; ROC curves and SPSS software were used for the assessment of the diagnostic value of circACTG1. Overall survival related to expression was evaluated by the Kaplan-Meier survival curve and the log rank test using GEPIA. Data were expressed as mean ± SD. *p* values < 0.05 were considered as statistical significance.

## 3. Results

circACTG1 was significantly amplified in HCC primary tissues and cell lines, correlated with worse prognosis.

**Figure 1:**
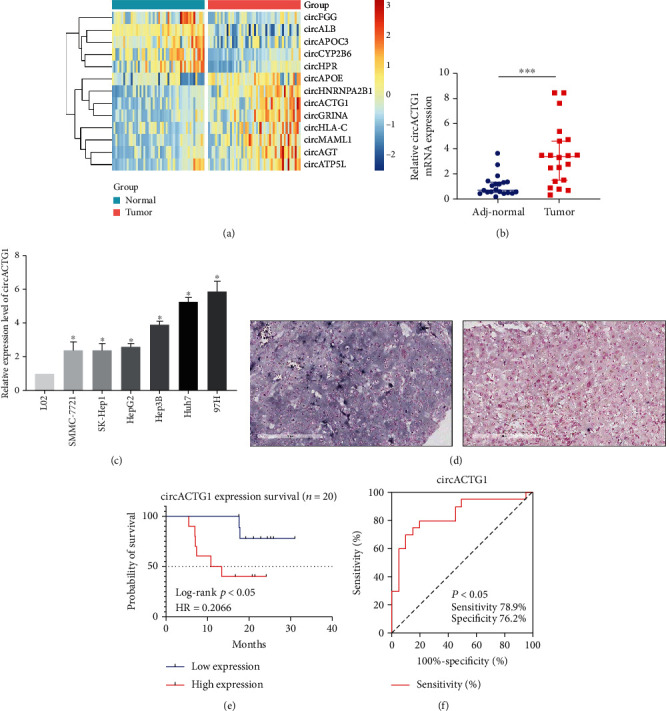
Overexpressed circACTG1 in HCC primary tissues and cell lines showed a collection to worse prognosis. (a) Different expression of circRNAs in normal and tumor group. (b) circACTG1 mRNA expression level in adjacent normal (adj-normal) and tumor tissues. (c) circACTG1 mRNA expression level in cell lines. (d) In situ hybridization (ISH) by digoxin biotin antibody in adjacent normal tissues and tumor tissues. (e) Overall survival (OS) time curve of 20 patients. (f) Receiver operating characteristic (ROC) curve analysis of circACTG1.

Firstly, RNA sequence was performed, and it was found that the differential expression ratio of circACTG1 was the highest in HCC tissues (*N* = 3) compared to adjacent normal tissues (*N* = 3, *p* < 0.05) (Figure 1(a)). Next, we utilized 20 cases of HCC and adj-normal tissues, respectively, to detect the expression of their mRNA levels of circACTG1, and found that it was significantly elevated in tumor tissues comparatively (*N* = 40, *p* < 0.001) (Figure 1(b)). At the same time, mRNA levels of circACTG1 were detected on the cell lines, and it displayed that all HCC cell lines showed high expression, among which 97H and Huh7 cells had the highest expression multiple (*p* < 0.05) (Figure 1(c)). In keeping with mRNA expression, digoxin biotin antibody was applied to determine circRNA level in tissues, and the results also showed higher circACTG1 expression in HCC tissues comparatively by in situ hybridization (ISH) (*N* = 20, *p* < 0.05) (Figure 1(d)). Further, we analyzed the overall survival time of these 20 patients, suggesting that the higher circACTG1 expression group had a significantly poorer prognosis (*p* < 0.05) (Figure 1(e)). ROC curve was conducted to evaluate circACTG1 diagnostic performance in HCC. The area under the curve (AUC) of circACTG1 was 0.84 (95% CI: 0.7116 to 0.9684) (*p* = 0.0002) (Figure 1(f)).

### 3.1. Knockdown of circACTG1 Inhibited HCC Cell Proliferation, Invasion, and Migration

**Figure 2:**
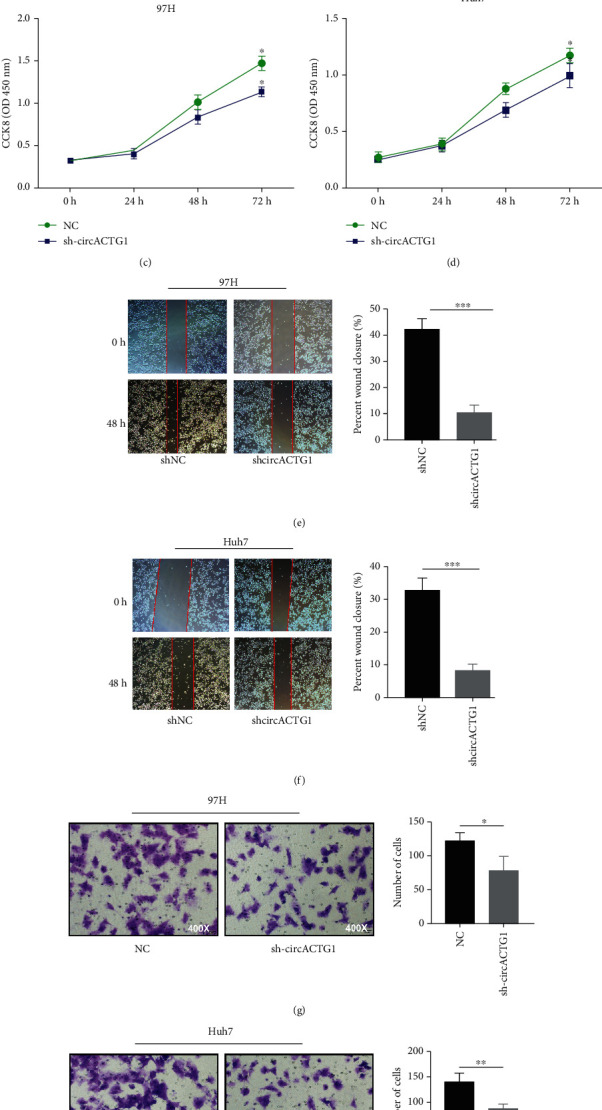
Knockdown of circACTG1 inhibited 97H and Huh7 cells proliferation, migration, and invasion. (a and b) Knockdown of circACTG1. (c–h) Abilities of proliferation (c and d), migration (e and f), and invasion (g and h) in 97H and Huh7 cells with circACTG1 knockdown were tested by CCK8 assay, wound healing assay, and transwell assay, respectively.

To investigate the underlying function of circACTG1 in HCC, circACTG1 expression was restrained in 97H and Huh7 cells via using shRNA plasma (*p* < 0.01) (Figures 2(a) and 2(b)), and our study revealed that circACTG1 knockdown inhibited two cell proliferations markedly at 48 h and 72 h (*p* < 0.05) (Figures 2(c) and 2(d)). In keeping with cell proliferation, circACTG1 knockdown also observably suppressed 97H and Huh7 cell invasion (*p* < 0.05) and migration (*p* < 0.001) (Figures 2(e)–2(h)). These results implied that circACTG1 is an oncogene that promoted HCC progression.

### 3.2. circACTG1 Acted as a miR-940 Sponge in HCC

**Figure 3:**
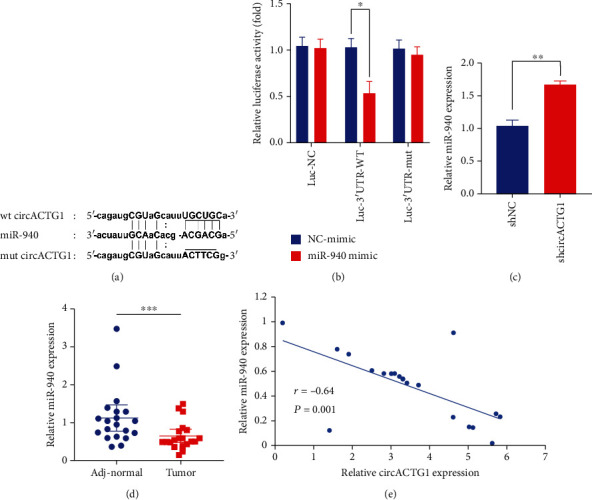
circACTG1 played as a miR-940 sponge in HCC. (a) circACTG1 may act as a miR-940 sponge as demonstrated by bioinformatics analysis predicted by circBase. (b) miR-940 mimic could markedly downregulate the luciferase activity of circACTG1. (c) circACTG1 knockdown significantly promoted the expression level of miR-940. (d) Expression of miR-940 in adjacent normal and tumor tissues. (e) Correlation of circACTG1 and miR-940 by Spearman's correlation coefficient analysis.

It is reported that circRNAs mainly participate as miRNA sponge. To further study the downstream regulation mechanism of circACTG1, the circBase database is utilized to predict possible targets. In our study, bioinformatics analysis demonstrated that circACTG1 may play as a miR-940 sponge (Figure 3(a)). The miR-940 mimic signally decreased Luc-3′UTR-WT (wt circACTG1) luciferase activity but had no effect on Luc-NC and Luc-3′UTR-mut (mut circACTG1) (*p* < 0.05) (Figure 3(b)). qPCR results also demonstrated that circACTG1 knockdown actively promoted miR-940 expression (*p* < 0.01) (Figure 3(c)). Meanwhile, significantly lower miR-940 expression was detected in HCC tissues than control tissues (*p* < 0.001) (Figure 3(d)). Spearman's correlation coefficient displayed a negative correlation between circACTG1 and miR-940 (*r* = −0.64, *p* = 0.001) (Figure 3(e)). All values showed that circACTG1 acted as a sponge of miR-940 in HCC.

### 3.3. circACTG1 Function in 97H Cells with circACTG1 Knockdown Was Rescued by miR-940

**Figure 4:**
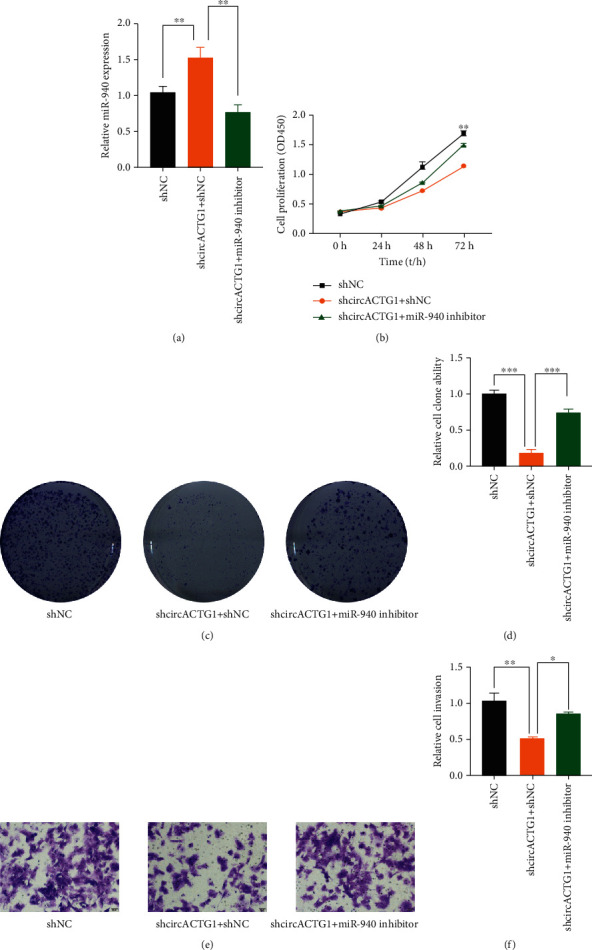
miR-940 rescued the function of circACTG1 in 97H cells with circACTG1 knockdown. (a) circACTG1 knockdown could significantly promoted the expression of miR-940, while it was partially inhibited when combined with miR-940 inhibitor. (b) Proliferation abilities of 97H cells with circACTG1 knockdown or combined with miR-940 inhibitor were tested by CCK8 assay. (c) Proliferation abilities of 97H cells with circACTG1 knockdown or combined with miR-940 inhibitor were detected by clone formation assay. (d) Invasion abilities of 97H cells with circACTG1 knockdown or combined with miR-940 inhibitor were detected by transwell assay.

To detect the mediate effect of miR-940 on circACTG1 knockdown, 97H cells with the highest circACTG1 expression were selected. In the first place, 97H cells with circACTG1 knockdown was transfected with or without miR-940 inhibitor, and it turned out that circACTG1 knockdown could significantly promote the expression of miR-940, which was partially inhibited when combined with miR-940 inhibitor (*p* < 0.01) (Figure 4(a)). CCK-8 assay displayed that circACTG1 knockdown restrained 97H cell proliferation, while this situation was restored by miR-940 inhibitor (*p* < 0.01) (Figure 4(b)). Clone formation assay and transwell assay also suggested that circACTG1 knockdown decayed the proliferation (*p* < 0.001) and invasion (*p* < 0.05) of 97H cells, whereas miR-940 inhibitor rescued this effect (Figures 4(c)–4(g)).

### 3.4. The AKT-mTOR Pathway Was Activated by circACTG1/miR-940/RIF1 Axis

**Figure 5:**
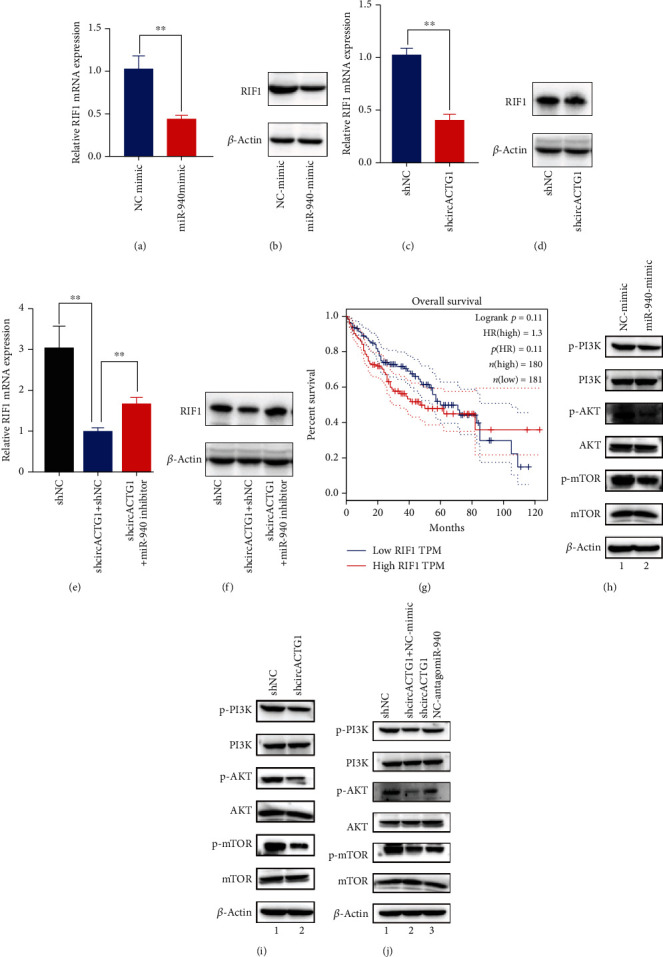
The AKT-mTOR pathway was activated by circACTG1/miR-940/RIF1 axis. (a and b) Expression of RIF1 after transfecting miR-940 mimic. (c and d) Expression of RIF1 after transfecting shcircACTG1. (e and f) Expression of RIF1 with circACTG1 knockdown or combined with miR-940 inhibitor. (g) Overall survival of RIF1 expression. (h–j) AKT-mTOR pathway molecule expression after transfecting miR940 mimic (h), shcircACTG1 (i), and shcircACTG1 and miR-940 inhibitor (j).

The results of bioinformatics analysis showed that RIF1 was a target of miR-940. We assessed RIF1 level after miR-940 overexpression, showing an obviously decreased RIF1 mRNA level after overexpressing miR-940 (*p* < 0.01) (Figure 5(a)). The protein level and mRNA level of RIF1 were consistent (Figure 5(b)), and both significantly reduced after silencing circACTG1 (*p* < 0.01) (Figures 5(c) and 5(d)), while in circACTG1-knockdown 97H cells which were transfected with the miR-940 inhibitor, RIF1 protein and mRNA expression was rescued (*p* < 0.01) (Figures 5(e) and 5(f)). Using The Cancer Genome Atlas data, the overall survival curve analysis displayed a consistent result that high RIF1 level was negatively associated with HCC patient survival rate (Figure 5(g)). Further experiments found that phosphorylation levels of mTOR and AKT decayed by miR-940 overexpression or circACTG1 knockdown (Figures 5(h) and 5(i)) but restored after inhibiting miR-940 expression in 97H cells with circACTG1 knockdown (Figure 5(j)). It revealed that circACTG1 regulated HCC biological function via the miR-940.

### 3.5. circACTG1 Promoted Tumorigenicity by Targeting miR-940 *In Vivo*

**Figure 6:**
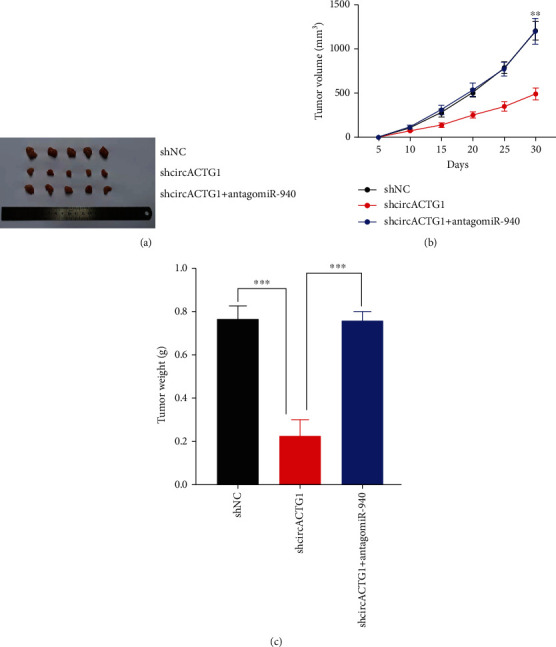
circACTG1 promoted tumorigenicity by targeting miR-940 in vivo. (a) 97H cells with circACTG1 knockdown (shcircACTG1) or normal control (shNC) were injected into the left and right dorsal flanks of nude mice. (b and c) Tumor size (b) and weight (c) were measured after 30 days.

To validate the findings *in vitro*, we injected 97H cells transfected with circACTG1 knockdown (shcircACTG1) or normal control (shNC) into nude mice on the left and right dorsal flanks, respectively. circACTG1 knockdown markedly inhibited tumor growth (*p* < 0.01) (Figures 6(a) and 6(b)) and reduced the tumor weight (*p* < 0.01) (Figure 6(c)) in subcutaneous xenograft models. Tumorigenic activity of circACTG1 was restored when an antagonist of miR-940 was added to 97H cells with circACTG1 knockdown.

## 4. Discussion

Over recent years, HCC incidence rate has been rising. Despite the continuous progress of medical technology, the survival rate of 5 years is still not optimistic [17]. In addition to radical surgery, other traditional treatments such as radiotherapy and chemotherapy are not sufficiently effective for HCC patients. Moreover, lots of liver cancer patients are already in the middle and late stages when they are diagnosed. Therefore, it is urgent to find new therapeutic target or biomarkers to improve the early diagnosis and therapy of HCC [18, 19]. Previously, circRNA was found to regulate the progression of various tumors (e.g., gastric cancer, lung cancer, and colorectal cancer) [20–22]. In our study, we first detected differentially expressed circRNAs in normal and tumor tissues and targeted circACTG1 as our research object. We discovered that circACTG1 expression was notably increased in HCC tissues and cell lines compared to control group and that patients with high circACTG1 expression had worse prognosis. To further clarify the functions of circACTG1, we transfected cell lines with knockdown circACTG1 and detected the changes in proliferation, migration, and invasion ability. It is reported that circRNAs usually sponge targeted miRNAs to play malignant biological behavior. Therefore, we used circBase database to predict the possible interaction sites between miR-940 and circACTG1, which was affirmed by the double luciferase reporter assay. Subsequently, we detected the miR-940 expression in the aforementioned samples and conducted Pearson's correlation analysis with circACTG1. Also, rescue experiment was performed with miR-940 inhibitor; the results all confirmed that circACTG1 binds miR-940.

Emerging evidence indicates that miRNAs could also bind to downstream target genes and inhibit their protein expression[23–26]. We identified RIF1 as the target of miR-940 by bioinformatics analysis, and consistent with this, patients with high RIF1 expression also had a worse prognosis. In the genesis and development of tumors, the upstream target genes produce cascade effects through signaling pathways, resulting in phenotypic changes. The AKT-mTOR pathway was proven to be activated by circACTG1/miR-940/RIF1 axis. Finally, we carried out experiments on nude mice *in vivo*, which strengthened the results in previous experiments *in vitro.*

## 5. Conclusion

In conclusion, our findings found that circACTG1 promotes HCC tumorigenesis as an oncogenic factor. The oncogenic function of circACTG1 was mediated by sponging to miR-940 via regulating AKT/mTOR signaling. High circACTG1 expression predicts poor prognosis for HCC patients. It is expected to become a key tumor marker and target for the diagnosis and cure of liver cancer in the future.

## Figures and Tables

**Table 1:** Sequence of primers used in the study.

Gene	Forward (5′-3′)	Reverse (5′-3′)
has_circ_0046144 (circACTG1)	CCCTTGGTATGGAATCTTGCG	CTCCTTCTGCATCCTGTCGG
*β*-Actin	GGCTGTATTCCCCTCCATCG	CCAGTTGGTAATGCCATGT
RIF1	CTCAGTATAGTCAGGAAGAGCCT	TCAGCCATACCACAGTCTTCCG
U6	AGAGAAGATTAGCATGGCCCCTG	ATCCAGTGCAGGGTCCGAGG
has_miR-940	AAGGCAGGGCCCCCG	GAACATGTCTGCGTATCTC

## Data Availability

The data that support the findings of this study are available from the corresponding author upon reasonable request.
